# Overweight and obesity increase breast cancer risk in postmenopausal women: a meta-analysis of 38 observational studies

**DOI:** 10.1007/s11357-026-02257-0

**Published:** 2026-04-14

**Authors:** Piercarlo Del Console, Stefania Catalano, Balázs Győrffy

**Affiliations:** 1 Department of Pharmacy, Health and Nutritional Sciences, Via P. Bucci, University of Calabria, Arcavacata Di Rende (CS), 87036 Cosenza, Italy; 2 Centro Sanitario, Via P. Bucci, University of Calabria, Arcavacata Di Rende (CS), 87036 Cosenza, Italy; 3 Clinical Laboratory Unit, A.O. “Annunziata”, Cosenza, Italy; 4 Department of Bioinformatics, Semmelweis University, 1094 Budapest, Hungary; 5 Institute of Transdisciplinary Discoveries, Medical School, University of Pecs, 7624 Pecs, Hungary; 6 Cancer Biomarker Research Group, Institute of Molecular Life Sciences, Hungarian Research Network, Magyar Tudósok Körútja 2, 1117 Budapest, Hungary

**Keywords:** Adiposity, BMI, Epidemiology, Meta-analysis, Systematic review, Menopausal status

## Abstract

Obesity is a recognized host-related factor associated with increased breast cancer (BC) risk—the most common malignancy and leading cause of cancer-related death among women. Menopausal status plays a critical role in this association due to hormonal and metabolic differences that may influence the impact of excess weight on carcinogenesis. This meta-analysis investigates the relationship between overweight, obesity, and BC risk, stratified by menopausal status. A systematic search of PubMed, Web of Science, and Cochrane Central Register of Controlled Trials databases (1987–2025) was conducted to identify observational studies examining the association between overweight, obesity, and BC risk. Odds ratios (ORs) and 95% confidence intervals (CIs) were extracted and pooled using a random-effects model. Subgroup analyses were performed for premenopausal and postmenopausal women. Publication bias was assessed through funnel plots and Egger’s test. Thirty-eight independent studies were included, totaling 115,870 women. In postmenopausal women, both overweight (OR = 1.31; 95% CI 1.13–1.53) and obesity (OR = 1.43; 95% CI 1.25–1.63) were significantly associated with increased BC risk. Among premenopausal women, the associations were weaker and not statistically significant for either overweight (OR = 1.04; 95% CI 0.83–1.3) or obesity (OR = 1.11; 95% CI 0.89–1.38). Funnel plots showed no publication bias. Overweight and obesity significantly increase BC risk in postmenopausal women, but not in premenopausal women. These findings highlight the role of menopausal status and support the need for weight management strategies, particularly in postmenopausal women, to reduce obesity-related breast cancer risk.

**Supplementary Information:**

The online version contains supplementary material available at 10.1007/s11357-026-02257-0.

## Introduction

Breast cancer (BC) is the most common malignancy and the leading cause of cancer-related mortality among women worldwide [1]. It encompasses a heterogeneous group of tumors characterized by distinct morphologies, molecular profiles, therapeutic responses, relapse risks, and survival outcomes. Pathological classification remains central to clinical management, guiding treatment strategies based on key prognostic and predictive biomarkers, including the hormone receptors estrogen receptor α (ERα) and progesterone receptor (PR), the human epidermal growth factor receptor 2 (HER2), and the Ki-67 proliferation marker [2]. Notably, ERα-positive tumors accounts for approximately 70–80% of all BC cases where endocrine-based therapies, particularly selective estrogen receptor modulators (SERMs) in premenopausal women, and aromatase inhibitors (AIs) in postmenopausal women play a pivotal role in both adjuvant and recurrent treatment settings [3].

BC risk is influenced by a plethora of non-modifiable factors including, genetic predispositions (e.g., BRCA1 and BRCA2 mutations), ethnicity, reproductive history, breast tissue density, prior BC history, previous radiation therapy, age, and menopausal status [4]. In contrast, modifiable risk factors include body weight, physical activity, alcohol consumption, chemical exposure, and smoking [4–7].

Obesity, a complex and multifaceted condition, is an established risk factor for a wide range of diseases, including cardiovascular events, diabetes, and several types of cancers, including BC [8–12]. Clinically, obesity is commonly assessed by calculating the body mass index (BMI). According to the World Health Organization (WHO), a normal BMI is defined as 18.5–24.9 kg/m^2^, overweight as 25–29.9 kg/m^2^, and obesity as ≥ 30 kg/m^2^ or higher, further classified into grade I (30–34.9 kg/m^2^), grade II (35–39.9 kg/m^2^), and grade III (≥ 40 kg/m^2^). In recent decades, the global prevalence of obesity has risen sharply, with one in eight people now classified as obese, marking a twofold increase in adults and a fourfold increase in adolescents since 1990 [13]. In Europe, obesity prevalence is lowest in younger adults (16–24 years) and highest in those aged 65–74 years, with socioeconomic disparities affecting prevalence. In particular, lower obesity rates were observed among people with higher levels of education, although men with secondary or non-tertiary education exhibited higher rates [14].

Epidemiological studies suggest that the impact of obesity on BC differs according to menopausal status. While obesity is consistently associated with an increased risk of postmenopausal BC, findings in premenopausal women remain controversial [15–17]. Beyond BMI, weight gain during adulthood, independent of baseline BMI, has been associated with an increased risk of postmenopausal BC, likely mediated by age-related hormonal and metabolic changes [18]. Understanding the magnitude of this association is essential for informing prevention strategies and public health policies. This meta-analysis aims to quantify the association between overweight, obesity and BC risk, with a particular focus on menopausal status. By analyzing multiple independent studies, we attempt to quantify the risk of BC associated with different levels of excess body weight.

## Methods

This systematic review and meta-analysis investigated the impact of excess body weight, including being overweight or obese, on the risk of breast cancer in premenopausal and postmenopausal women. The review and meta-analysis were conducted and reported in accordance with the relevant items of the 2020 Preferred Reporting Items for Systematic Reviews and Meta-Analyses (PRISMA) statement (Supplementary Table S1 and S2) [19], and in accordance with recent consensus guidelines for meta-analyses in biomedical research [20].

### Search strategy

**Fig. 1:**
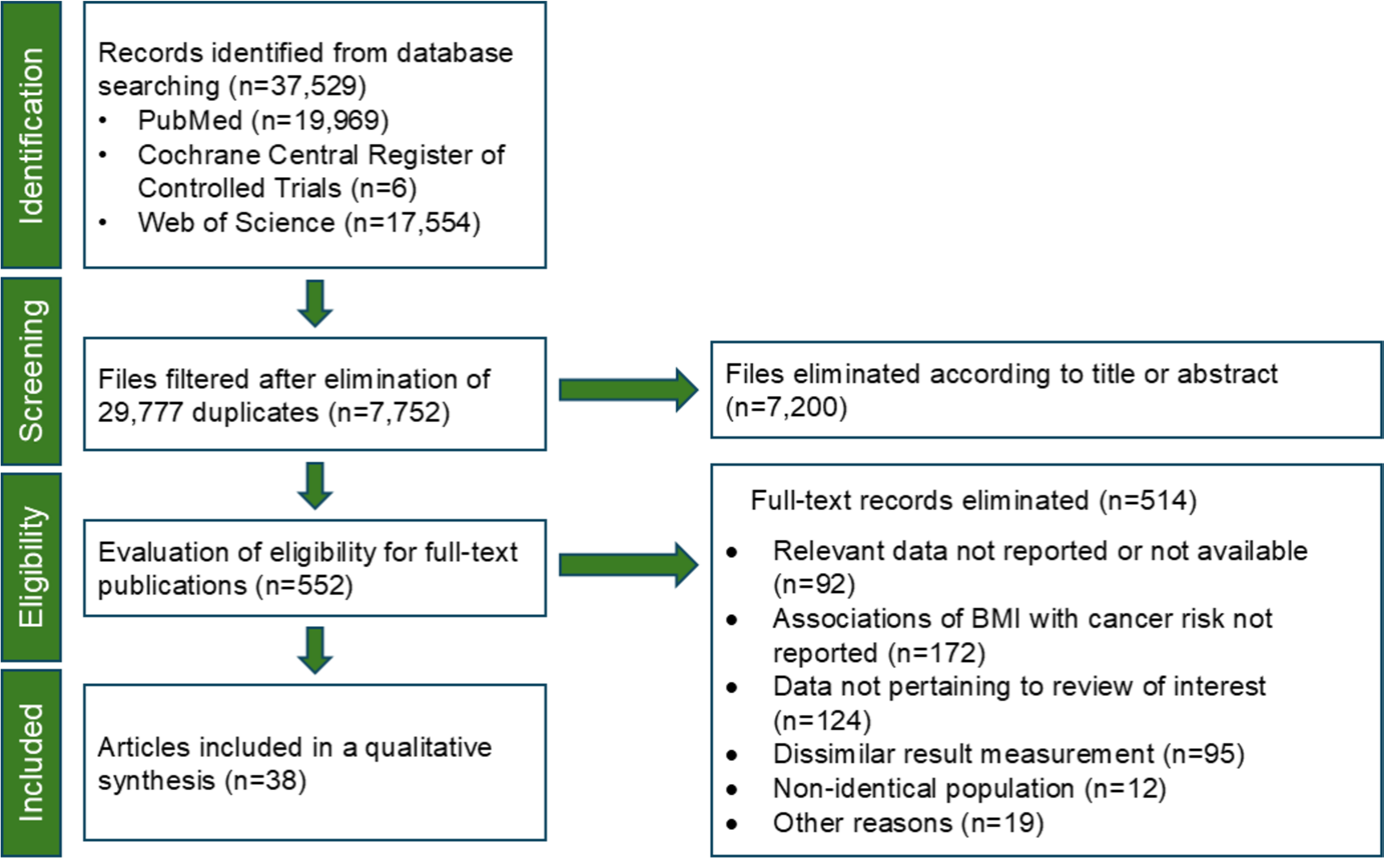
Flow diagram of the article selection process

We conducted a systematic search of the PubMed, Web of Science and Cochrane Central Register of Controlled Trials (CENTRAL) databases from 1987 to 2025 to identify studies examining the associations between overweight and obesity and the risk of breast cancer (Fig. 1). The search terms used included “breast cancer” in combination with “body mass index,” “overweight,” and “obesity.” We also collected studies from previously published meta-analyses [15, 21–29].

### Study eligibility assessment

After screening the publication title and abstract, the eligibility of the studies was assessed by using the full text of the article. Studies not published in full text, such as conference abstracts and letters to the editor, as well as those focusing on survival and cancer mortality instead of incidence, were excluded. We included studies that classified BMI ranges based on the World Health Organization criteria as having overweight (25–29.9 kg/m^2^) and obesity (≥ 30 kg/m^2^)—as only studies adhering to these thresholds were included, no additional harmonization was required.

### Determining the overall effect

Statistical analyses were performed using the web application available at https://metaanalysisonline.com [30]. A random-effects model was applied to calculate pooled odds ratios (ORs) with corresponding 95% confidence intervals (CI). To visualize individual study results and the overall effect estimate, forest plots were generated, providing a graphical summary of effect sizes and data dispersion. The heterogeneity among the studies was assessed using the chi-squared test and the *I*^2^ index to quantify variability not attributable to random chance.

Potential publication bias was evaluated through funnel plots, which illustrate the relationship between effect sizes and study precision. Additionally, Egger’s test was performed to statistically assess the presence of publication bias.

### Subcohort analysis settings

To provide a comprehensive understanding, we conducted the statistical analyses across several specific settings. Initially, we performed two different combined analyses to explore the risk of breast cancer according to BMI (overweight and obese) independently of menopausal states. Subsequently, we carried out separate analyses for pre- and postmenopausal cohorts to determine menopausal status-related breast cancer risk.

## Results

### Included studies and quality assessment

The methodological quality of the 38 included studies was assessed using the Newcastle–Ottawa Scale (NOS) for observational studies. The included studies comprised observational designs and were evaluated across the three NOS domains—selection, comparability, and outcome. The resulting scores ranged from moderate-high to high (8–9 out of a maximum of 9), indicating an overall good methodological quality and supporting the validity of the meta-analytic results.

### Breast cancer risk in overweight premenopausal women

**Fig. 2:**
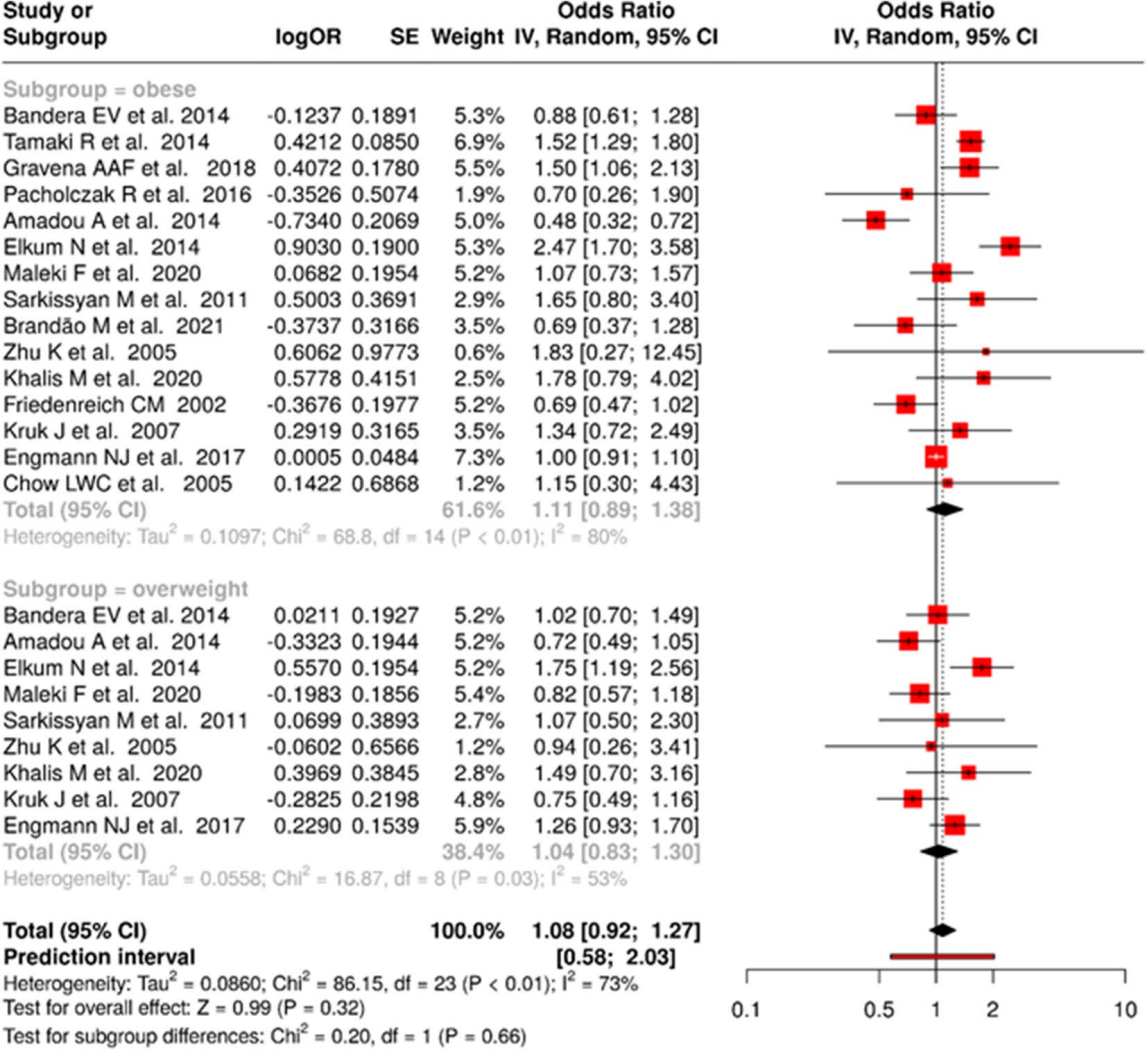
Meta-analysis of observational studies linking obesity, overweight and breast cancer incidence in premenopausal women. Abbreviations: CI, confidence interval; OR, odds ratio; IV, inverse variance; SE, standard error

A total of nine studies were analyzed [31–39] (see complete list in Table 1). Based on the analysis performed using random effects model with inverse variance method to compare the OR, there is no statistical difference, the summarized OR is 1.04 with a 95% confidence interval of 0.83—1.3 and the test for overall effect does not show a significant effect. We detected a significant heterogeneity (*p* = 0.03), suggesting inconsistent effects in magnitude and/or direction; the *I*^2^ value indicates that 53% of the variability among studies arises from heterogeneity (Fig. 2).

**Table 1:** Characteristics of studies included in the meta-analysis of premenopausal women overweight or obese and breast cancer risk

Authors	Year	OR	Lower CI	Upper CI	Subcohort	*n *cases	*n* controls	References
Bandera EV et al.	2014	1.02	0.7	1.49	Overweight	150	142	[30]
Amadou A et al.	2014	0.72	0.49	1.05	Overweight	182	200	[31]
Elkum N et al.	2014	1.74	1.19	2.56	Overweight	86	148	[32]
Maleki F et al.	2020	0.82	0.57	1.18	Overweight	222	244	[33]
Sarkissyan M et al.	2011	1.1	0.5	2.3	Overweight	29	29	[34]
Zhu K. et al.	2005	0.93	0.26	3.41	Overweight	10	14	[35]
Khalis M. et al.	2020	1.49	0.7	3.16	Overweight	64	64	[36]
Kruk J. et al.	2007	0.75	0.49	1.16	Overweight	90	154	[37]
Engmann N. J. et al.	2017	0.99	0.93	1.7	Overweight	1456	15,123	[38]
Bandera E. V. et al.	2014	0.89	0.61	1.28	Obese	217	237	[30]
Khan A. et al.	2017	0.14	0.02	0.77	Obese	72	59	[110]
Gravena A. A. F. et al.	2018	1.64	1.06	2.13	Obese	12	27	[40]
Pacholczak R. et al.	2016	0.7	0.26	1.9	Obese	12	25	[41]
Amadou A. et al.	2014	0.48	0.32	0.72	Obese	133	202	[31]
Elkum N. et al.	2014	2.47	1.7	3.58	Obese	111	135	[32]
Maleki F. et al.	2020	1.07	0.73	1.57	Obese	211	196	[33]
Sarkissyan M. et al.	2011	1.7	0.8	3.4	Obese	53	37	[34]
Brandão M. et al.	2021	0.69	0.37	1.28	Obese	25	89	[42]
Zhu K. et al.	2005	1.84	0.27	12.45	Obese	4	7	[35]
Khalis M. et al.	2020	1.78	0.79	4.02	Obese	54	46	[36]
Friedenreich CM	2002	0.69	0.47	1.02	Obese	102	119	[43]
Kruk J. et al.	2007	1.34	0.72	2.49	Obese	33	44	[37]
Engmann N. J. et al.	2017	1	0.91	1.1	Obese	616	7192	[38]
Chow L. W. C. et al.	2005	1.32	0.3	4.43	Obese	5	8	[44]

**Fig. 3:**
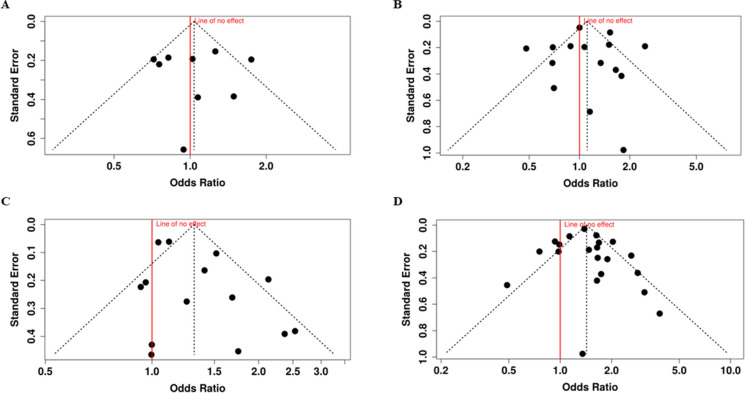
Funnel plots indicate no potential publication bias in the four different setting analyzed, including observational studies for BC risk in premenopausal overweight (**A**) and obese (**B**) women and in postmenopausal overweight (**C**) and obese (**D**) women

The funnel plot does not validate a probable publication bias. Egger’s test excludes any funnel plot asymmetry (intercept, − 0.03, 95% CI − 3–2.94; t, − 0.017; *p*-value, 0.987) (Fig. 3A).

### Breast cancer risk in obese premenopausal women

The analysis involved a total of 15 studies [31–45]. No statistically significant difference was observed using the random effects model. The pooled OR is 1.11, with a 95% confidence interval of 0.89 to 1.38. The test for overall effect does not show a significant effect. At the same time, significant heterogeneity was observed (*p* < 0.01), indicating inconsistent effects in magnitude and/or direction. The *I*^2^ value suggests that 80% of the variability across studies is due to heterogeneity rather than random variation (Fig. 2). The funnel plot does not prove a likely publication bias. Egger’s test dismisses the presence of funnel plot asymmetry (intercept, 0.19; 95% CI, − 1.54–1.92; t, 0.212; *p*-value, 0.836) (Fig. 3B).

Overall, the pooled analysis of both overweight and obese subcohorts does not support a significant association between BMI and breast cancer risk in premenopausal women. The combined OR is 1.08 (95% CI, 0.92–1.27), indicating no statistically significant increase in risk. However, substantial heterogeneity was observed (*p* < 0.01), with an *I*^2^ value of 73%, suggesting that most of the variability among studies is due to heterogeneity rather than random chance.

### Breast cancer risk in overweight postmenopausal women

**Fig. 4:**
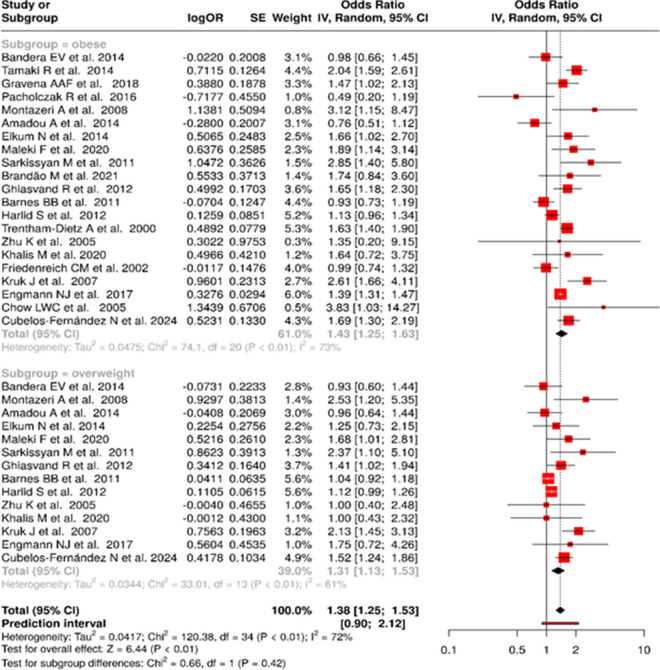
Meta-analysis of observational studies linking obesity, overweight, and breast cancer incidence in postmenopausal women. Abbreviations: CI, confidence interval; OR, odds ratio; IV, inverse variance; SE, standard error

A total of 14 studies were scrutinized [31–39, 46–50] (see complete list in Table 2). According to the investigation results using random effects model with inverse variance method to compare the OR, a statistical difference can be detected; the summarized OR is 1.31 with a 95% confidence interval of 1.13–1.53. The assessment for overall effect displays a *p* value below 0.05. Notably, a significant heterogeneity was also found (*p* < 0.01), pointing at inconsistent effects in magnitude and/or direction. An *I*^2^ value of marks that 61% of the inconsistency among the cohorts stems from heterogeneity rather than chance alone (Fig. 4).

**Table 2:** Characteristics of studies included in the meta-analysis of postmenopausal women overweight or obese and breast cancer risk

Authors	Year	OR	Lower CI	Upper CI	Subcohort	*n* cases	*n* controls	References
Bandera E. V. et al.	2014	0.93	0.6	1.44	Overweight	131	132	[30]
Montazeri A. et al.	2008	2.53	1.2	5.35	Overweight	51	47	[45]
Amadou A. et al.	2014	0.96	0.64	1.44	Overweight	239	213	[31]
Elkum N. et al.	2014	1.25	0.73	2.15	Overweight	70	42	[32]
Maleki F. et al.	2020	1.69	1.01	2.81	Overweight	142	140	[33]
Sarkissyan M. et al.	2011	2.3	1.1	5.1	Overweight	37	40	[34]
Ghiasvand R. et al.	2012	1.39	1.02	1.94	Overweight	208	193	[46]
Barnes B. B. et al.	2011	1.04	0.92	1.18	Overweight	1309	622	[47]
Harlid S. et al.	2012	1.12	0.99	1.26	Overweight	311	508	[48]
Zhu K. et al.	2005	0.99	0.4	2.48	Overweight	18	20	[35]
Khalis M. et al.	2020	0.99	0.43	2.32	Overweight	49	59	[36]
Kruk J. et al.	2007	2.13	1.45	3.13	Overweight	221	218	[37]
Engmann N. J. et al.	2017	1.23	0.72	4.26	Overweight	4476	43,937	[38]
Cubelos-Fernández N. et al.	2024	1.52	1.24	1.86	Overweight	402	395	[49]
Bandera E. V. et al.	2014	0.98	0.66	1.45	Obese	304	276	[30]
Tamaki R. et al.	2014	2.03	1.59	2.61	Obese	181	109	[39]
Gravena A. A. F. et al.	2018	1.5	1.02	2.13	Obese	23	60	[40]
Pacholczak R. et al.	2016	0.49	0.2	1.19	Obese	44	38	[41]
Montazeri A. et al.	2008	3.21	1.15	8.47	Obese	42	29	[45]
Amadou A. et al.	2014	0.75	0.51	1.12	Obese	257	314	[31]
Elkum N. et al.	2014	1.66	1.02	2.7	Obese	137	62	[32]
Maleki F. et al.	2020	1.9	1.14	3.14	Obese	181	152	[33]
Sarkissyan M. et al.	2011	2.9	1.4	5.8	Obese	91	81	[34]
Brandão M. et al.	2021	1.73	0.84	3.6	Obese	26	48	[42]
Ghiasvand R. et al.	2012	1.61	1.18	2.3	Obese	141	119	[46]
Barnes B. B. et al.	2011	0.93	0.73	1.19	Obese	105	259	[47]
Harlid S. et al.	2012	1.13	0.96	1.34	Obese	311	508	[48]
Trentham-Dietz A. et al.	2000	1.6	1.4	1.9	Obese	1286	1051	[50]
Zhu K. et al.	2005	1.35	0.2	9.15	Obese	4	4	[35]
Khalis M. et al.	2020	1.64	0.72	3.75	Obese	64	51	[36]
Friedenreich C. M. et al.	2002	0.99	0.74	1.32	Obese	199	191	[43]
Kruk J. et al.	2007	2.62	1.66	4.11	Obese	122	97	[37]
Engmann N. J. et al.	2017	1.39	1.31	1.47	Obese	2493	23,321	[38]
Chow L. W. C. et al.	2005	3.82	1.03	14.27	Obese	10	3	[44]
Cubelos-Fernández N. et al.	2024	1.68	1.3	2.19	Obese	188	168	[49]

The funnel plot does not show a potential publication bias. Egger’s test excludes a funnel plot asymmetry (intercept, 1.19; 95% CI, − 0.04–2.43; t, 1.19; *p*-value, 0.082) (Fig. 3C).

### Breast cancer risk in obese postmenopausal women

A total of 21 cohorts were investigated [31–51]. Applying a random effects model with the inverse variance method revealed a statistically significant difference, with a pooled OR of 1.43 (95% confidence interval, 1.25–1.63). The test for overall effect delivers a significant *p* value below 0.05. A significant heterogeneity was spotted (*p* < 0.01), signifying variable effects in extent and/or direction. An *I*^2^ value of 73% indicates that the variability among the cohorts is primarily due to heterogeneity rather than random chance (Fig. 4).

The funnel plot does not indicate a potential publication bias. Egger’s test does not support the presence of funnel plot asymmetry (intercept, 0.31; 95% CI, − 0.85–1.46; t, 0.521; *p*-value, 0.608) (Fig. 3D).

Overall, the pooled analysis indicates a significant association between overweight, obesity and BC risk in postmenopausal women, with a combined OR of 1.38 (95% CI, 1.25–1.53). This suggests that higher body weight is correlated with an increased risk of BC in this population. However, substantial heterogeneity was observed (*p* < 0.01, *I*^2^ = 72%), indicating that variability among studies is largely due to differences in study characteristics rather than random variation.

### Breast cancer risk independent of menopausal status

When analyzing the entire cohort combined, independent of menopausal status, a total of 17 studies examined the association between overweight and BC risk [33, 35, 36, 49, 52–64]. Using a random effects model, no statistically significant difference was observed. The pooled OR was 1.07 (95% CI, 0.90–1.20), and the test for overall effect did not indicate a significant association. A total of 22 studies investigated the relationship between obesity and BC risk [33, 35, 36, 42, 49, 52–68]. In this analysis, a statistically significant association was found. The pooled OR was 1.28 (95% CI, 1.05–1.56), with the test for overall effect yielding a *p*-value below 0.05. Significant heterogeneity was present (*p* < 0.01), suggesting inconsistent effects in magnitude and/or direction. The *I*^2^ value indicated that 87% of the observed inconsistency among studies was attributable to heterogeneity rather than random variation. No evidence of publication bias was observed in the funnel plot, and Egger’s test did not indicate funnel plot asymmetry (intercept, 0.86; 95% CI, − 1.15 to 2.88, t = 0.842, *p* = 0.41).

## Discussion

Our meta-analysis found that overweight and obesity increase the risk of postmenopausal breast cancer by 31% and 43%, respectively. However, we did not find a statistically significant association in premenopausal women.

The rising global prevalence of obesity—“globesity”—poses a major public health and economic burden. Beyond its link to diet-related noncommunicable diseases, obesity is increasingly recognized as a key factor in cancer biology. Studies indicate its association with higher overall and cancer-specific mortality, accounting for nearly one-third of new cancer cases and influencing recurrence across various malignancies [12, 69, 70]. During obesity, white adipocytes undergo hypertrophy and hyperplasia, leading to metabolic alterations characterized by increased levels of free fatty acids (FFA), cholesterol, elevated triglycerides, reduced high-density lipoprotein (HDL), and elevated blood glucose and insulin resistance. Compensatory hyperinsulinemia further enhances mitogenic and anti-apoptotic signaling via the insulin/Insulin Like Growth Factor 1 (IGF-1) axis, fostering a tumorigenic microenvironment [71–74]. Indeed, IGF-1 binding to its receptor IGF-1R, activates downstream Phosphatidylinositol 3-kinase/Protein Kinase B (PI3K/AKT) and Mitogen-activated protein kinases (MAPK) pathways, driving cell proliferation, survival, and metastasis [75].

Obesity-reprogrammed adipose tissue secretes inflammatory cytokines [e.g., interleukin-1 (IL-1), IL-6, tumor necrosis factor alpha (TNF-α)], adipokines, and nanoscale lipid-bilayer enclosed particles referred to as extracellular vesicles (EVs). These factors collectively contribute to cancer progression [71, 76]. Among the adipokines, leptin and adiponectin exert opposing effects. Leptin, whose levels correlate with increased adiposity, promotes cell proliferation by modulating cell cycle regulators, such as Cyclin-Dependent Kinase 2 (CDK2) and cyclin D1 [77], enhances angiogenesis and inflammation through the Janus Kinase 2 (JAK)/Signal Transducer and Activator of Transcription 3 (STAT3), PI3K/AKT and MAPK signaling pathways [78, 79]. Leptin may also sustain cell–cell communication by regulating EV biogenesis through a post-transcriptional induction of Tumor Susceptibility Gene 101 (Tsg101), a key protein involved in EV generation [80]. These leptin-related EVs can then favor metabolic rewiring in recipient cells [81]. Conversely, adiponectin, an anti-inflammatory adipokine, enhances insulin sensitivity and inhibits proliferation via AMP-activated protein kinase (AMPK) activation [82, 83]. An imbalanced leptin-to-adiponectin ratio in obesity fosters a pro-tumorigenic milieu [84]. Moreover, adipocyte-derived EVs enhanced growth, motility, invasion, stem cell-like properties of breast cancer cells, promoting lung metastatic colonization [85]. More interestingly, serum EVs reflected a differential cargo in relation to a patient’s BMI, which could impact breast cancer biology [86].

Breast cancer risk is strongly influenced by age and menopausal status due to differences in estrogen bioavailability. In premenopausal women, ovarian estrogen production predominates, while cytochrome P-450 aromatase facilitates the peripheral conversion of C19 steroids in both the adrenal gland and ovary, minimizing the impact of adipose-derived estrogen [87]. This may explain why no statistically significant association was observed between overweight, obesity, and BC risk in this subcohort. In addition, the impact of obesity on premenopausal BC risk varies according to the subtype. Shain and colleagues conducted a retrospective analysis of 3767 BC patients and demonstrated that triple negative breast cancer (TNBC) was the most common subtype in obese premenopausal women compared to the normal weight counterpart. Moreover, patients with higher BMI were found to have more advanced and aggressive tumors at the time of diagnosis [88]. Conversely, several studies report that in young women a higher BMI is linked to an increased risk of developing ER+/PR+ BC rather than TNBC, suggesting a strong association between BMI and HR+ BC [89, 90]. Taken together, these findings suggest that the relationship between BMI and BC risk in premenopausal women is highly subtype-dependent. Opposing associations across tumor subtypes, such as a potential positive association with TNBC and a different or even inverse relationship with HR+ disease, may contribute to the missing overall effect observed in pooled analyses. This highlights the importance of considering tumor heterogeneity when interpreting epidemiological associations and suggests that subtype-specific analyses are essential to fully elucidate the role of obesity in premenopausal breast cancer risk. In conclusion, in premenopausal women, the interpretation of estrogen level data is more challenging due to their limited availability and the considerable fluctuations of endogenous estrogen throughout the menstrual cycle.

In contrast, after menopause, ovarian estrogen production ceases, and estrogens are primarily synthesized in adipose tissue through the aromatization of androstenedione into estrone [91]. In an obese context, an excess in estrogen production can promote a pro-tumorigenic environment via cytokines such as IL-6, TNF-α, and C-reactive protein (CRP) [92, 93]. Interestingly, it has been demonstrated that for overweight and obese postmenopausal women, HR + BC risk is 1.5 to 2 times higher [94, 95], along with a poor prognosis, endocrine resistance and greater chance of BC recurrence as compared to lean counterpart [12, 88, 96]. Circulating factors present in the serum of obese postmenopausal women have been shown to amplify the crosstalk between non genomic ER signaling and PI3K/AKT and Raf pathways, thereby promoting breast cancer progression and treatment failure [97]. Leptin has been demonstrated to mimic the effects of ERα transactivation in ER + BC cell line, which includes a decrease in ER levels in terms of mRNA and protein, and an increase of estrogen-dependent gene pS2 involved in endocrine therapy resistance [98, 99]. Furthermore, hormone replacement therapy (HRT) may modulate the relationship between BMI and BC risk. Several studies suggest that HRT use, particularly combined estrogen-progestin therapy, is associated with an increased risk of HR + BC, potentially amplifying the tumor-promoting effects of obesity in postmenopausal women [100, 101]. Moreover, obesity-related hyperinsulinemia and chronic inflammation could still influence BC risk through mechanisms independent of estrogen signaling, particularly in ER- subtypes [102]. In this context, Thiagarajan and colleagues proposed a model in which constitutive activation of STAT3 during obesity can act as a signaling hub with possible implications for the transition and maintenance of TNBC stem cells due to an activated leptin receptor (LEPRb) pathway [103].

Beyond increasing breast cancer incidence, obesity also worsens prognosis, influencing survival, and recurrence rates in both pre- and postmenopausal women [96, 104, 105]. A comprehensive meta-analysis by Chan and colleagues examined data from 82 follow-up studies, encompassing both premenopausal and postmenopausal women with BC. The findings revealed that obesity is associated with poorer OS and BC specific survival, regardless of menopausal status [105]. Furthermore, a recent meta-analysis assessed the impact of obesity on survival outcomes in patients with different cancers, including BC. The study found that recurrence and overall mortality was increased in patients with obesity and BC with a hazard ratio (HR) of 1.14 (95% CI, 1.10–1.19) and 1.23 (95% CI, 1.15–1.32) respectively, when compared to non-obese patients [70].

Given the strong link between obesity and BC outcomes, weight gain emerges as a crucial modifiable risk factor [106, 107]. A population-based case control study of BC demonstrated that weight gain was not associated with breast cancer risk in premenopausal women but significantly increased the risk in postmenopausal women, particularly for weight gain occurring between first pregnancy and menopause (OR 1.91, 95% CI 1.26–2.88). The association was more pronounced for ER/PR-positive tumors and among women with greater central adiposity [108]. Moreover, a strong association between adult weight gain and postmenopausal ER + BC risk is reported in several studies [107, 109].

To counteract the negative impact of obesity on BC risk and survival, weight management interventions, including weight loss and dietary modifications, have been proposed. An analysis conducted on 61,335 women without prior BC revealed that during a mean follow-up of 11.4 years, women who achieved a sustained weight loss of ≥ 5% had a significantly lower risk of postmenopausal BC compared to those with stable weight [100]. Additionally, the role of dietary composition has been explored in a cohort of 48,835 postmenopausal women, where adherence to a low-fat dietary pattern enriched with fruits and vegetables was associated with both a lower incidence of BC and a reduction in BC-specific mortality [110]. These findings suggest that weight control, when combined with a balanced diet, may act as a modifiable factor in BC prevention and prognosis, underscoring the importance of lifestyle-based interventions in high-risk populations.

However, this study is not without limitations. The observed heterogeneity among included studies indicates variability in study populations, methods, and potential confounders that may influence the results. First, differences in geographic regions and ethnicity may reflect variability in genetic background, lifestyle factors, and baseline BC risk. Second, HRT use was inconsistently reported and adjusted for, despite its known impact on breast cancer risk, particularly in postmenopausal women. In addition, variability in BMI assessment methods, including self-reported versus measured anthropometric data, may have introduced measurement bias. Furthermore, differences in tumor subtype composition across studies, particularly regarding HR + versus TNBC, may partially explain the lack of association observed in premenopausal women. Differences in fat distribution may also contribute to the observed heterogeneity, as premenopausal women exhibit distinct adipose tissue patterns that could differentially influence tumorigenesis. Additionally, hormonal factors, including estrogen fluctuations, may modulate breast cancer susceptibility in premenopausal women. The interaction of obesity with other risk factors, such as alcohol consumption, smoking, and metabolic dysfunction, warrants further investigation. Although funnel plot analysis and Egger’s test did not indicate significant publication bias, the potential presence of unpublished negative studies remains a concern.

In conclusion, this meta-analysis highlights a significant incremental association between overweight, obesity and increased postmenopausal BC risk. Public health policymakers and healthcare providers should emphasize obesity prevention and management as a fundamental approach to reducing cancer risk. Future studies incorporating tumor subtype-specific analyses will be essential to better define the differential impact of obesity across BC subtypes, particularly in premenopausal women.

## Supplementary Information

Below is the link to the electronic supplementary material.

ESM 1 (DOCX 18.1 KB)

ESM 2 (DOCX 21.2 KB)

## Data Availability

All data generated and/or analyzed during this study are included in this article. Requests for materials should be addressed to B.G.; gyorffy.balazs@yahoo.com.

## Declarations

The review was not registered. Protocol was not reported.
